# Discovery of Functional Alternatively Spliced *PKM* Transcripts in Human Cancers

**DOI:** 10.3390/cancers13020348

**Published:** 2021-01-19

**Authors:** Xiangyu Li, Woonghee Kim, Muhammad Arif, Chunxia Gao, Andreas Hober, David Kotol, Linnéa Strandberg, Björn Forsström, Åsa Sivertsson, Per Oksvold, Hasan Turkez, Morten Grøtli, Yusuke Sato, Haruki Kume, Seishi Ogawa, Jan Boren, Jens Nielsen, Mathias Uhlen, Cheng Zhang, Adil Mardinoglu

**Affiliations:** 1 Science for Life Laboratory, KTH—Royal Institute of Technology, SE-17165 Stockholm, Sweden; xiangyu.li@scilifelab.se (X.L.); woonghee.kim@scilifelab.se (W.K.); muhammad.arif@scilifelab.se (M.A.); hober@kth.se (A.H.); david.kotol@scilifelab.se (D.K.); lstrandb@kth.se (L.S.); bjorn.forsstrom@scilifelab.se (B.F.); asa.sivertsson@scilifelab.se (Å.S.); per.oksvold@scilifelab.se (P.O.); mathias.uhlen@scilifelab.se (M.U.); 2 Department of Chemistry and Molecular Biology, University of Gothenburg, SE-41296 Gothenburg, Sweden; chunxia.gao@chem.gu.se (C.G.); grotli@chem.gu.se (M.G.); 3 Department of Medical Biology, Faculty of Medicine, Atatürk University, Erzurum 25240, Turkey; hturkez@atauni.edu.tr; 4 Department of Pathology and Tumor Biology, Institute for the Advanced Study of Human Biology (WPI-ASHBi), Kyoto University, Kyoto 606-8501, Japan; satoy-uro@h.u-tokyo.ac.jp (Y.S.); sogawa-tky@umin.ac.jp (S.O.); 5 Department of Urology, Graduate School of Medicine, The University of Tokyo, Tokyo 113-8654, Japan; kume@kuc.biglobe.ne.jp; 6 Centre for Hematology and Regenerative Medicine, Department of Medicine, Karolinska Institute, SE-17177 Stockholm, Sweden; 7 Department of Molecular and Clinical Medicine, University of Gothenburg, Sahlgrenska University Hospital, SE-41345 Gothenburg, Sweden; Jan.Boren@wlab.gu.se; 8 Department of Biology and Biological Engineering, Chalmers University of Technology, SE-41296 Gothenburg, Sweden; nielsenj@chalmers.se; 9 BioInnovation Institute, DK-2200 Copenhagen N, Denmark; 10 Key Laboratory of Advanced Drug Preparation Technologies, School of Pharmaceutical Sciences, Ministry of Education, Zhengzhou University, Zhengzhou 450001, China; 11 Centre for Host-Microbiome Interactions, Faculty of Dentistry, Oral & Craniofacial Sciences, King’s College London, London SE1 9RT, UK

**Keywords:** alternative splicing, cancer, *PKM*, transcriptomics

## Abstract

**Simple Summary:**

Pyruvate kinase muscle type (*PKM*) is a key enzyme in glycolysis and is a mediator of the Warburg effect in tumors. The association of *PKM* with survival of cancer patients is controversial. In this study, we investigated the associations of the alternatively spliced transcripts of *PKM* with cancer patients’ survival outcomes and explained the conflicts in previous studies. We discovered three poorly studied alternatively spliced *PKM* transcripts that exhibited opposite prognostic indications in different human cancers based on integrative systems analysis. We also detected their protein products and explored their potential biological functions based on in-vitro experiments. Our analysis demonstrated that alternatively spliced transcripts of not only *PKM* but also other genes should be considered in cancer studies, since it may enable the discovery and targeting of the right protein product for development of the efficient treatment strategies.

**Abstract:**

Pyruvate kinase muscle type (*PKM*) is a key enzyme in glycolysis and plays an important oncological role in cancer. However, the association of *PKM* expression and the survival outcome of patients with different cancers is controversial. We employed systems biology methods to reveal prognostic value and potential biological functions of *PKM* transcripts in different human cancers. Protein products of transcripts were shown and detected by western blot and mass spectrometry analysis. We focused on different transcripts of *PKM* and investigated the associations between their mRNA expression and the clinical survival of the patients in 25 different cancers. We find that the transcripts encoding PKM2 and three previously unstudied transcripts, namely ENST00000389093, ENST00000568883, and ENST00000561609, exhibited opposite prognostic indications in different cancers. Moreover, we validated the prognostic effect of these transcripts in an independent kidney cancer cohort. Finally, we revealed that ENST00000389093 and ENST00000568883 possess pyruvate kinase enzymatic activity and may have functional roles in metabolism, cell invasion, and hypoxia response in cancer cells. Our study provided a potential explanation to the controversial prognostic indication of *PKM*, and could invoke future studies focusing on revealing the biological and oncological roles of these alternative spliced variants of *PKM*.

## 1. Introduction

Pyruvate kinase muscle type (*PKM*) is the most-studied isoform of pyruvate kinase and catalyzes the final step in glycolysis [1]. It is one of the key mediators of the Warburg effect and plays a pivotal role in controlling tumor metabolism. It has been reported that the mRNA and protein expression of *PKM* is strongly associated with the survival of cancer patients, but the direction of the correlation was contradictory since both activation and inhibition of this enzyme have been suggested for effective treatment of the cancer patients [2]. In the Human Pathology Atlas [3], high expression of *PKM* is significantly (log-rank *p*-value < 0.05) associated with the unfavorable prognoses in liver hepatocellular carcinoma (LIHC), pancreatic adenocarcinoma (PAAD), head and neck squamous cell carcinoma (HNSC), and lung adenocarcinoma (LUAD), whereas it is also associated with favorable prognoses in kidney renal clear-cell carcinoma (KIRC), skin cutaneous melanoma (SKCM), stomach adenocarcinoma (STAD), and thyroid carcinoma (THCA). Thus, mRNA expression of *PKM* has ambiguous indication of patients’ survival in different cancer types.

The oncological roles of differentially spliced transcripts of *PKM* including PKM1 and PKM2, which are mutually exclusive exons 9 and 10 [4], have been previously investigated. PKM1 constitutively exists as the active tetramer to generate adenosine triphosphate (ATP) to supply cellular energy. However, PKM2 can exist as tetramer or dimer, and the tetramer form is allosterically activated by the glycolytic metabolite fructose-1,6-biphosphate (FBP) [5,6]. FBP tightly binds to tetrameric PKM2 [7] and the release of FBP causes PKM2 to switch from tetramer to dimer [8]. Tetrameric PKM2 exhibits high catalytic activity, which is associated with ATP synthesis and catabolic metabolism [9], while dimeric PKM2 has low catalytic activity and is the less active state of PKM2. 

PKM2 activity is also regulated by post-translational modification such as phosphorylation [10,11,12,13], acetylation [14,15], oxidation [16], and sumoylation [17], which promote aerobic glycolysis or tumorigenesis. In addition, nuclear PKM2 also can work as a transcriptional regulator to activate HIF-1α [18], β-catenin [11,19], MEK5 [20], and Oct-4 [21], which promotes cell proliferation and tumor development. It has been reported that over-expression of PKM1/2 isoforms promotes tumorigenesis or induces poor prognoses of patients in multiple cancers [19,22,23,24,25,26,27,28,29,30,31,32,33] whereas PKM1 expression in place of PKM2 inhibits tumor cell proliferation [34,35]. Moreover, it has been reported that methylation or deletion of PKM2 promotes tumor progression in liver cancer, breast cancer, and medulloblastoma [36,37,38,39]. Therefore, the function of alternative splicing products of *PKM* in tumor oncogenesis and progression remains controversial.

Due to alternative splicing, there are 14 known isoforms of the *PKM*, of which PKM1 and PKM2 are well studied isoforms. To our knowledge, the roles of other protein products of *PKM* apart from PKM1 and PKM2 have not been studied. In this study, we focused on 14 different transcripts of *PKM* and systematically investigated the biological functions of each transcript as well as their association with the clinical outcomes in 25 different cancer types.

## 2. Results

### 2.1. The Alternatively Spliced Protein Coding Transcripts of Pyruvate Kinase Muscle Type (PKM)

**Figure 1:**
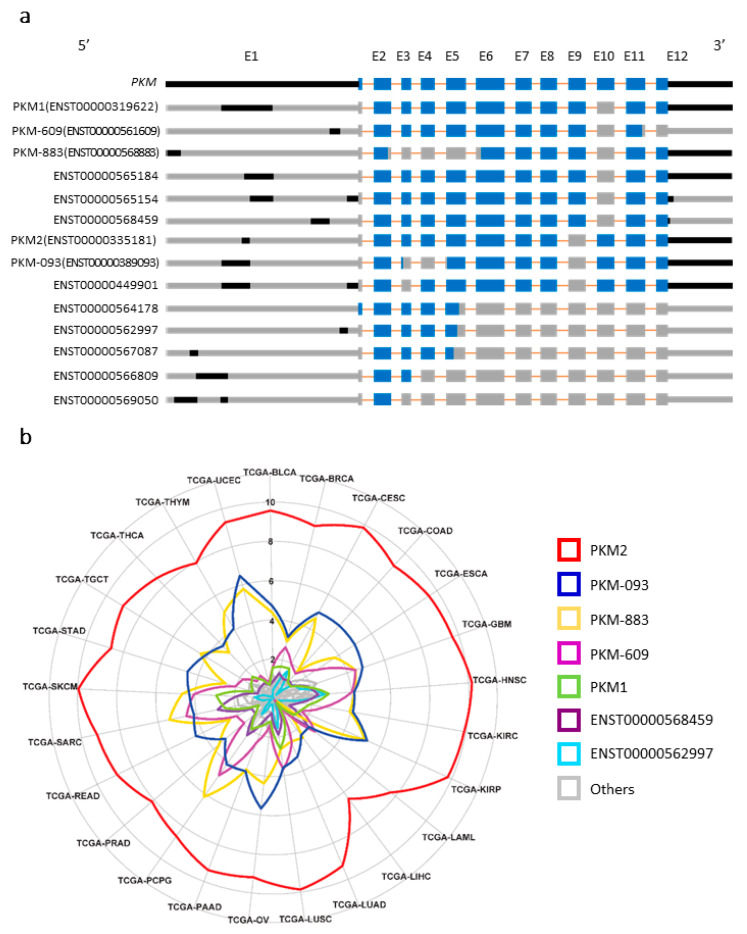
Exon–intron structure of pyruvate kinase muscle type (PKM) transcripts and their corresponding expression levels in cancer and normal tissues. (**a**) Exon–intron structure of PKM transcripts. Blue color denotes translated region of exon. Orange color denotes intron. Black color denotes untranslated region of exon. Gray color denotes the missing sequence. (**b**) The expression levels of PKM transcripts in 25 human cancers from the Cancer Genome Atlas Program (TCGA). The transcripts per kilobase million (TPM) values were on log 2 scale.

*PKM* is composed of 12 exons according to the reference genome Ensembl Version 83 (GENCODE Version 24; Figure 1a; Table S1). Both of the two commonly studied transcripts of *PKM*, ENST00000319622 (encoding PKM1) and ENST00000335181 (encoding PKM2), are composed by 11 exons where 10 of them are shared. The most important difference between PKM1 and PKM2 is their specificity on exon 9 and 10, where PKM1 includes the former and PKM2 includes the latter. In addition to the transcripts of PKM1 and PKM2, there are another 12 known transcripts of *PKM* and with different lengths of nucleotide sequences and exon combinations. As shown in Figure 1a, ENST00000561609 (PKM-609), ENST00000568883 (PKM-883), ENST00000565184, ENST00000565154, and ENST00000568459 are composed of different subsequences from PKM1, as well as ENST00000389093 (PKM-093) and ENST00000449901 are composed of different subsequences from PKM2. ENST00000564178 has its special translated region in exon E1. The other transcripts are very short subsequences of PKM1/PKM2.

Next, we retrieved mRNA expression of these 14 transcripts in 25 different cancer types in The Cancer Genome Atlas (TCGA; Table S2). As shown in Figure 1b, we found that seven transcripts had mRNA expression (average TPM) > 5 in at least one cancer type, including PKM1, PKM2, PKM-093, PKM-609, PKM-883, ENST00000562997, and ENST00000568459. Among them, PKM2 shows the highest expression level in all the cancer types followed by PKM-093 and PKM-883, and it represents ~95% of *PKM* mRNA expression. 

### 2.2. The Prognostic Effect of PKM at the Transcript Level

**Figure 2:**
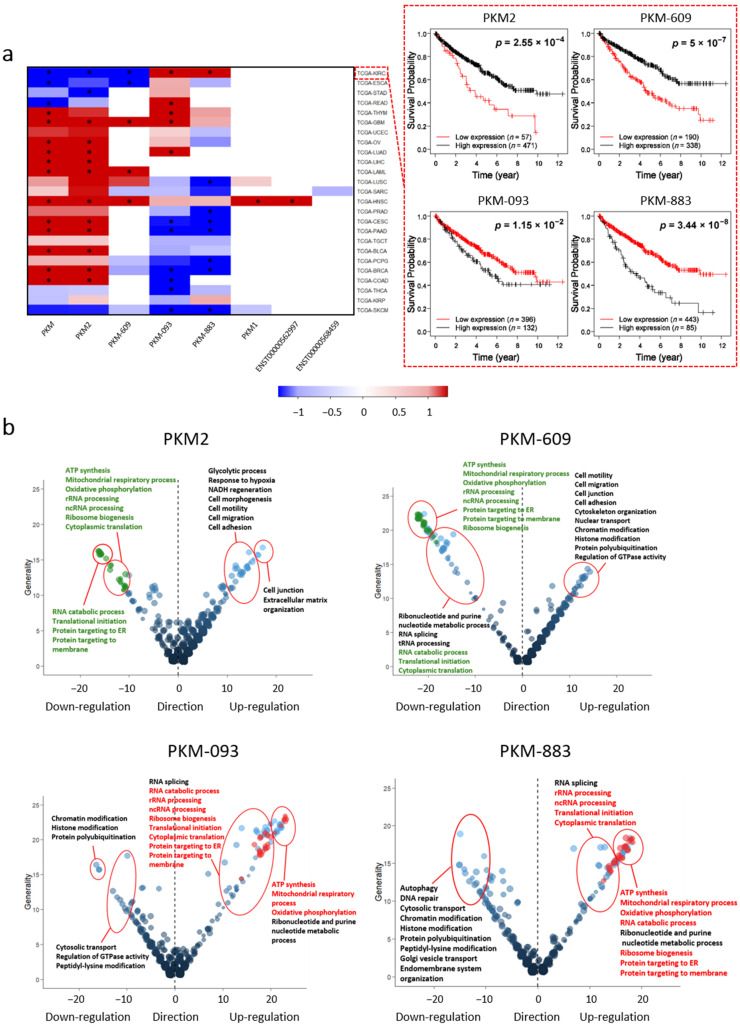
Prognostic and functional analysis of PKM and its alternatively spliced transcripts in TCGA cancers. (**a**) Heat map of the log-rank *p*-values (on the negative log 10 scale) of PKM and seven transcripts (average TPM > 5) in 25 cancer-types. Six of these transcripts are significantly associated with patients’ survival outcome in at least one cancer. The Kaplan–Meier plots for kidney renal clear-cell carcinoma (KIRC) was exemplified. Asterisk means log-rank test *p* < 0.05. (**b**) Bubble plot showing the common enriched gene ontology (GO) terms among the 25 cancer-types in the TCGA. False discovery rate (FDR) < 0.001 was used to identify the significantly enriched GO terms. Bubble sizes represent numbers of genes associated to the biological function in a specific GO term; the x and y axes indicate the directions and generalities of the GO terms. Generality is defined by the number of cancers with differentially expressed genes (DEGs) associated with each transcript; direction is defined by the number of cancers with their upregulated genes over-representing the GO function minus the number of cancers with downregulated genes over-representing the GO function. Note that only functions enriched with more than 10 cancers are shown. The red bubbles denote the commonly detected GO terms enriched with upregulated DEGs and the green bubbles denote the commonly detected GO terms enriched with downregulated DEGs.

We investigated the associations between the mRNA expression of *PKM* and its seven highly expressed transcripts with the patients’ survival outcomes based on the clinical survival metadata of patients in TCGA. We performed a Kaplan–Meier survival analysis for the patients by classifying the patients into two groups with high and low expression of the investigated transcript by optimally selecting a cutoff from the 10th to 90th expression percentiles yielding the lowest log-rank *p*-value as in our previous study [3] (Tables S3 and S4). As shown in Figure 2a, the mRNA expression of the *PKM* indicated opposite survival outcomes in different cancer types. At the transcript level, we found six of these transcripts, including PKM1, PKM2, PKM-609, PKM-093, PKM-883, and ENST00000562997, are significantly associated with patients’ survival outcome in at least one cancer. Among them, the mRNA expression of transcript encoding PKM2 exhibited a very similar prognostic indication to *PKM* in all cancer types since it dominates the mRNA expression of *PKM* (Table S2). Notably, we observed that the expression levels of transcript encoding PKM1, which has been associated with different cancer types [22,23,40,41] is only prognostic in HNSC. 

High expression of PKM2, PKM-609, PKM-093, and PKM-883 indicated opposite clinical survival outcome in different cancer types. This is exemplified by PKM-883, whose high expression indicates unfavorable survival in KIRC patients and favorable survival in lung squamous cell carcinoma (LUSC), prostate adenocarcinoma (PRAD), cervical squamous cell carcinoma and endocervical adenocarcinoma (CESC), PAAD, pheochromocytoma and paraganglioma (PCPG), breast invasive carcinoma (BRCA), and SKCM. We also found that the expression of *PKM* indicated an opposite survival outcome of patients compared to that of its transcripts in multiple cancer types. For example, PKM-883 exhibited the opposite prognostic indication compared to *PKM* in KIRC, CESC, PAAD, and BRCA (Figure 2a). Moreover, the high expression of different *PKM* transcripts may induce opposite prognoses in patients with the same cancer. This is exemplified in KIRC, where high expression of PKM2 and PKM-609 pair indicated favorable prognoses of patients and high expression of PKM-093 and PKM-883 pair indicated the opposite, and similar scenarios could be found in CESC, PAAD, BRCA, and colon carcinoma (COAD). 

### 2.3. Potential Biological Functions Associated with PKM Transcripts

We identified four different transcripts of *PKM* including PKM2, PKM-609, PKM-093, and PKM-883 which exhibited opposite prognostic effect in multiple cancer types. Hence, we investigated whether this opposite trend is also observed at the functional level in all cancer types. To systematically identify the functions of the four prognostic *PKM* transcripts, we identified the differentially expressed genes (DEGs) between patients with the top 25% high expression and bottom 25% low expression of each transcript in all cancers (FDR < 1.0 × 10^−5^). We performed a gene ontology (GO) enrichment analysis using the DEGs driven by each transcript, and summarized the results of the enriched GO terms for all transcripts in all cancer types (FDR < 0.001, Figure 2b and Table S5). As shown in Figure 2b, if a GO term is enriched with DEGs in multiple cancers, the directionality of the DEGs often follows the same direction. For example, as shown in Figure 2b, the DEGs identified by comparing high and low expression of PKM2 are enriched in extracellular matrix organization pathway in 16 cancers and are always associated with the upregulated genes. Our analysis indicated that although the prognostic effect of each transcript is different, the associated biological functions are conserved in different cancers. 

Next, we focused on GO terms that are consistently enriched in more than 10 cancers and conservatively associated with the corresponding transcript. We investigated the enriched GO terms associated with PKM2. As shown in Figure 2b, glycolytic process, hypoxia response, nicotinamide adenine dinucleotide hydride (NADH) regeneration pathways are enriched with upregulated genes in patients with high expression of PKM2. This is expected since it reflects the key enzymatic role in glycolysis of PKM2. On the other hand, ATP synthesis, mitochondrial respiratory process, oxidative phosphorylation pathways are enriched with downregulated genes, which probably indicates the shift from oxidative phosphorylation to glycolysis that is well known as the Warburg effect in cancer. Moreover, the upregulated genes were also enriched in pathways associated with the cell morphology such as cell motility, cell migration, cell adhesion, and cell junction pathways. These could be linked to the tumorigenesis role of PKM2 [11,24,25,42]. Interestingly, several RNA processing related pathways, translational initiation pathways, and pathways related to protein localization were also enriched with downregulated genes in cancer patients with high expression of PKM2. These pathways were rarely associated with the biological function of *PKM* in previous studies and appeared as commonly enriched GO terms in the same analysis for three other transcripts. Hence, studying alternative splicing processes of *PKM* in different cancers might provide further understanding about the potential role of *PKM* in cancer progression.

Then, we investigated the function of three other transcripts of *PKM* whose functions were not known. We found that many of the enriched GO terms associated with these three transcripts are similar to the GO terms associated with PKM2 (Figure 2b). However, the GO terms associated with the PKM-609 followed the same direction with those of PKM2, of which followed the opposite direction with the GO terms associated with both PKM-093and PKM-883. In total, 27 different GO terms, e.g., oxidative phosphorylation, translational initiation, and RNA catabolic processes, are enriched with genes that are downregulated with both PKM2 and PKM-609, and genes that are upregulated with both PKM-093 and PKM-883. 

We also compared the DEGs identified by comparing high and low expression of each transcript and observed similar results based on the directionality and overlap of the DEGs. For instance, in KIRC (Figure S1), we identified 3162 and 6592 DEGs (FDR < 1.0 × 10^−5^) when comparing the high and low expression of PKM2 and PKM-609, respectively, which both exhibited favorable prognostic indications. We found that the two sets of DEGs had a significant overlap (*n* = 2010; hypergeometric distribution test, *p* < 1.11 × 10^−16^) and the concordance score of these overlapped genes (using directionality of the DEGs) is 99.06%. We also identified 6541 and 6885 DEGs (FDR < 1.0 × 10^−5^) when comparing the high and low expression of PKM-093 and PKM-883, respectively, which both exhibited unfavorable prognostic effect in KIRC. Notably, we found that the overlap between the DEGs of the transcripts is 5469 (hypergeometric distribution test, *p* < 1.11 × 10^−16^) and the concordance score of the overlapped genes is 100%. On the other hand, we investigated the overlap between DEGs associated with the transcripts exhibiting the opposite prognostic effect and found that there is no statistically significant overlap. For instance, in KIRC, the concordance score of the overlapped DEGs between transcripts with opposite prognostic indications were between 0.10% and 20% (Table S6 and Figure S1). Similar scenarios were also observed in CESC, PAAD, BRCA, and COAD (Tables S7–S10). Our results suggested that both PKM2 and PKM-609 have similar biological functions that are opposite to those of PKM-883 and PKM-093.

### 2.4. Validation of the Prognostic Effect in Independent Kidney Renal Clear-Cell Carcinoma (KIRC) Cohort

**Figure 3:**
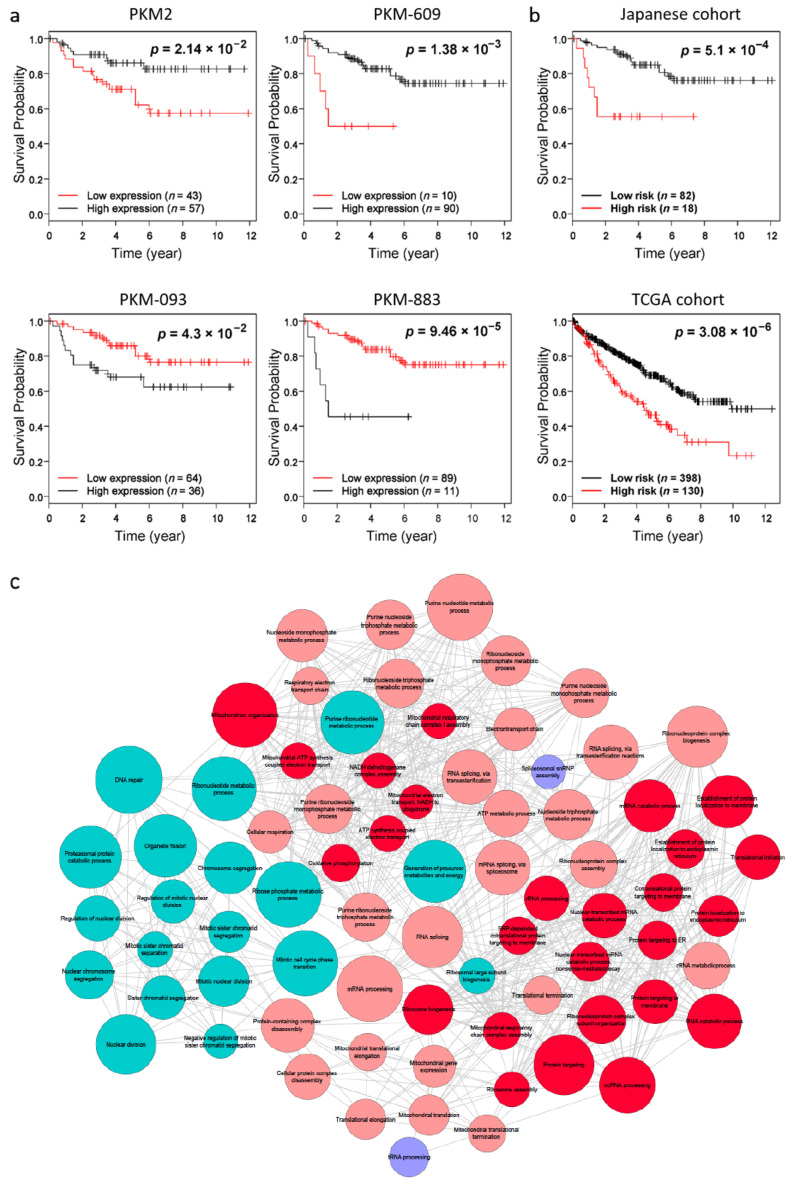
Validation of the prognostic effect and biological functions of the transcripts in an independent KIRC cohort. (**a**) The Kaplan–Meier plots for the samples classified by the high and low expression of transcripts including PKM2, PKM-609, PKM-093, and PKM-883 in Japanese cohort. (**b**) The Kaplan–Meier plots for the samples classified by the prognostic signature in TCGA and Japanese KIRC cohorts. (**c**) Network plot of enriched GO terms for DEGs between TCGA and Japanese KIRC cohorts. Sizes of the nodes are correlated with the corresponding total number of genes, and connections between the nodes indicate the significant overlaps (hypergeometric distribution test; FDR < 1.0 × 10^−^^5^) between the genes of the corresponding GO terms. Nodes in red, blue, and purple indicate GO terms that enriched in both cohorts, only in Japanese KIRC cohort, and only in TCGA KIRC cohort, respectively. The nodes highlighted in dark red indicate the common GO terms associated with all four transcripts reported in this study.

We performed a survival analysis for these four transcripts in 100 KIRC patients involved in an independent Japanese study [43]. As shown in Figure 3a, the high expression of both PKM2 and PKM-609 are significantly (log-rank *p* < 0.05) associated with the favorable survival of patients whereas the high expression of PKM-093 and PKM-883 are significantly associated with an unfavorable survival of patients. Our analysis indicated that PKM2 and PKM-609 are favorable prognostic transcripts and PKM-093 and PKM-883 are unfavorable prognostic transcripts in KIRC and it was similar to the results based on the TCGA KIRC cohort. Moreover, we employed univariate and multivariate Cox analysis to evaluate the prognostic value of these four transcripts (Table S11). In the univariate Cox analysis, all four transcripts exhibited significant association with patients’ overall survival in the TCGA cohort. Moreover, after taking the PKM2 as a covariate, PKM-093 and PKM-883 are still significantly prognostic in multivariate Cox analysis. Considering that PKM-609 and PKM-883 are more closed to PKM1 rather than PKM2 in terms of nucleotide sequence, we also took PKM1 as the covariate and performed multivariate Cox analysis. Our result showed that all four transcripts still exhibited significant prognostic effect.

To investigate whether these transcripts regulate the similar genes in two different cohorts, we compared the DEGs by comparing the expression of each transcript in patients with the top 25% high expression and bottom 25% low expression in Japanese and TCGA KIRC cohorts. For fair comparison, we selected the top 20% of the DEGs (*n* = 2694) in TCGA and Japanese cohorts and checked their overlap between genes. We found that the number of overlapped DEGs identified for PKM-609, PKM-093, and PKM-883 are 1370, 1499, and 1449 (hypergeometric distribution test, *p* < 1.11 × 10^−16^) and the concordance scores of the overlapped genes between the cohorts are 100%, 99.93%, and 99.86%, respectively (Table S12). Our analysis indicated that the biological functions associated with each of these three transcripts are highly conserved in an independent KIRC cohort. However, we found that the number of overlapping DEGs identified with the transcript PKM2 in both cohorts is relatively small (*n* = 546; hypergeometric distribution test, *p* ≈ 1) and the concordance score of the overlapped genes is 75.46%, which also indicates the differences between the two cohorts. Such differences may be explained by the dietary and racial differences between the two independent cohorts. 

### 2.5. Combined Prognostic Signature for KIRC

Based on the highly conserved prognostic effects of the *PKM* transcripts in two independent KIRC cohorts, there are likely to be different molecular subtypes among KIRC patients with opposite expression patterns of the transcripts highlighted in this study. Thus, we extracted a prognostic signature based on the expression value of these four transcripts (see Method). In brief, if more than half of the transcripts indicate an unfavorable prognosis, the patient is classified as high-risk and otherwise as low-risk. Using this rule, we observed significantly different overall survival (log-rank *p* < 0.01) between high- and low-risk groups in both TCGA and Japanese KIRC cohorts (Figure 3b). 

To investigate whether these two molecular subtypes identified in both cohorts exhibited similar biological differences, we extracted the top 20% most significant DEGs (*n* = 2694) between high-risk and low-risk groups in the TCGA and Japanese cohorts. The two lists of DEGs had significant overlap (*n* = 1516; hypergeometric distribution test, *p* < 1.11 × 10^−16^) and the concordance score of the overlapped genes was 100%. In addition, we identified 57 and 74 GO terms that are significantly enriched with upregulated genes (FDR < 1.0 × 10^−5^) in the high-risk group of the TCGA and Japanese cohorts, respectively (Figure 3c, Table S13). Interestingly, we found that 55 of these enriched GO terms are common in both cohorts and the molecular subtypes identified by our analysis have consistent biological differences. Moreover, 26 of the 27 GO terms that are significantly associated with the four transcripts (e.g., oxidative phosphorylation, translational initiation, and RNA catabolic process) are also among the overlapped enriched GO terms (Figure 2b). Our analysis indicated that the molecular subtypes are functionally related to the PKM2 and other three functional key transcripts of *PKM* identified in this study.

### 2.6. Discovery of the Protein Products of the Prognostic Transcripts

**Figure 4:**
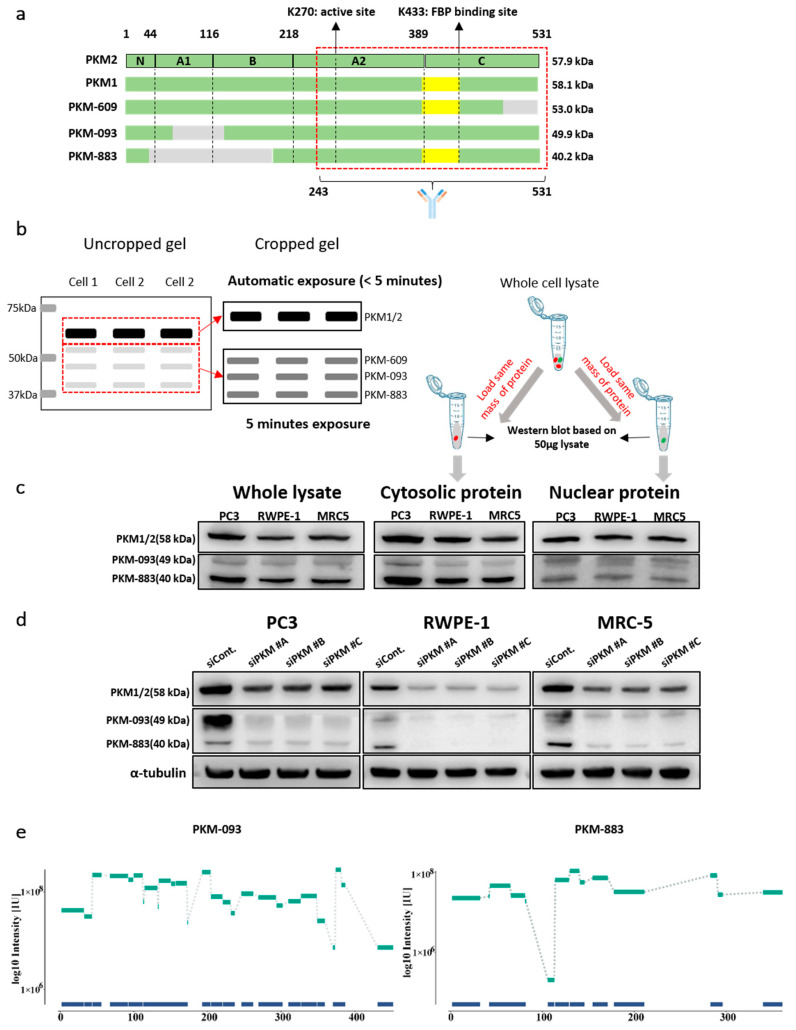
Protein products of the functional alternatively spliced PKM transcripts. (**a**) Alignment of amino acid sequence of the four functional different transcripts and the transcript encoding PKM1. The gray color denotes missing part compared to PKM2. The yellow color denotes a subsequence that is specific to PKM1. (**b**) Schematic diagram showing the different exposure time and gel cutting strategy to optimize the visualization of the different bands of PKM isoforms. The band for PKM1/2 is visualized via automatic exposure time in the full gel while the PKM-093 and PKM-883 bands are obtained after gel cropping and the exposure time is increased to 5 min to optimize the visualization. (**c**) Western blots for the proteins encoded by the transcript PKM-093 and PKM-883 in cytosol and nucleus with the same protein mass (see Method section). The red dot means cytosolic protein and the green dot means nuclear protein. (**d**) Western blots showing the protein level of PKM1/2 and protein products of PKM-093 and PKM-883 with siRNA and negative control. The exposure time and gel cutting strategy is same as (**c**). (**e**) Peptides detected using MS aligned with amino-acid sequence of respective transcript products on the *x*-axis and intensity on the *y*-axis.

To investigate and compare the protein products of the three novel transcripts including PKM-609, PKM-093, and PKM-883, whose functions were previously unknown compared to the function of PKM2, we first aligned their amino acid (AA) sequences (Table S14). We observed that PKM2 has the longest AA sequence with 531aa, followed by the protein products of PKM-609, PKM-093, and PKM-883, which are 485aa, 457aa, and 366aa, respectively. As shown in Figure 4a, we found that proteins of PKM-093 and PKM-883 miss a part of the A1 and B domains (59-132aa and 41-205aa) in PKM2, which may affect the formation of dimer [44]. We also found that the protein product of PKM-609 is shorter than PKM2, missing amino acid residues 486-531aa from PKM2 which is a part of the C-domain participating in the formation of tetramer [44]. This implied that the protein encoded by PKM-609 may have no tetrameric formation. In addition, there is part of the AA sequence, 389-433aa, of the protein product of PKM-609 and PKM-883 resembles PKM1 protein rather than PKM2. In this part, K433 is the FBP binding site in PKM2, which activates the association of monomer to form the tetrameric [6]. However, PKM1 does not bind FBP due to AA difference at the FBP binding pocket and it naturally exists as a stable tetramer that has high constitutive activity [45]. In addition, all protein products of the three transcripts have K270, which is the active site, binding to phosphoenolpyruvate (PEP). 

Furthermore, we constructed the homology models of PKM-609, PKM-093, and PKM-883 to obtain the protein structure information. When compared to the PKM2 structure, we found that the PKM-093 structure is missing the catalytic site for adenosine diphosphate (ADP) binding (59-132aa) and several AA residues, including R73, Q75, H78, G79, H80, E118, and R120 from the missing part are in close contact with ADP (Figure S2). Instead, the PKM-093 structure forms a newly ordered loop going straight through the ADP binding site, and whether this loop is able to coordinate the ADP binding is still unknown. On the other hand, we observed that the PEP and the FBP binding site in PKM-093 structure is fully maintained as in the PKM2 structure, and the tetramer binding interface is kept the same as in PKM2 structure. The protein product of PKM-609 shares exactly the same AA sequence as in PKM1, but misses the AAs from 486 to 531 in PKM1. By overlapping the structure of the protein product of PKM-609 with PKM1, we found that the protein maintains well defined ADP and PEP binding sites. However, the missing part constitutes part of the C-C binding interface (Figure S2). Therefore, whether the PKM-609 functions as a dimer or active tetramer needs further investigation. Comparing the protein of PKM-883 with PKM1, we observed that it misses a large part of both A and B domain as well as the whole N-terminal part. This led to a loss of a large part in the ADP binding site and the binding interface, whereas the PEP binding site in PKM-883 structure (Figure S2) was kept.

As we have shown that the AA sequence of the protein products of PKM-609, PKM-093, and PKM-883 resemble different parts of either PKM1 or PKM2, it is difficult to stratify them based on the AA sequence only. However, we observed that all of these transcripts have a different length of AA sequences, and different protein masses. The protein masses for PKM1 and PKM2 are 58.1 kDa and 57.9 kDa, respectively, while the mass for protein products of PKM-609, PKM-093, and PKM-883 are 53.0 kDa, 49.9 kDa, and 40.2 kDa, respectively. Therefore, we could separate these proteins based on their mass differences using sodium dodecyl sulfate (SDS) gel electrophoresis and evaluated them by western blot using one antibody that targeted their common sequence using a method developed in a previous study [46]. We selected an antibody whose targeting sequence is between the 243 to 531 AA of PKM2 to detect the other three transcripts, and as shown in Figure 4a we predict this antibody should also be able to bind to the other three transcripts since they share a large portion of AA sequence. As the expression of PKM-609, PKM-093, and PKM-883 are much lower than the expression of PKM2 and the antibody was not originally designed to target these three variants, we expected that the bands of PKM-609, PKM-093, and PKM-883 will have much less intensity. Thus, we used different exposure time and gel cutting strategy to optimize the visualization of the different bands in order to help to discover the predicted bands for PKM isoforms. As shown in Figure 4b, the band for PKM1/2 is visualized via automatic exposure procedure for the full gel while the PKM-609, PKM-093, and PKM-883 bands are obtained after gel cropping and the exposure time is increased to 5 min to optimize the visualization. As we expected, we found that there are different bands that appeared around 49 kDa and 40 kDa in the western blots (Figure 4c and Figure S3) in the whole lysate of the three different human cell lines, which is in very good agreement with the putative mass of protein products from PKM-093 and PKM-883, respectively. We also quantified the relative protein level of protein products from PKM-093 and PKM-883 compared to PKM1/2 based on the uncropped western blots image of whole lysate (Table S15) and found that they are similar to the relative mRNA level observed in tumor samples from TCGA (Table S2). In addition, we also extracted the cytosolic and nuclear protein to detect the two predicted isoforms by western blots (see Method section). We observed the two bands in the same location in the western blots of the cytosolic and nuclear proteins, which implies these two proteins could potentially play regulatory roles in cell nucleus as PKM2. 

Although we observed the bands that potentially represent the protein products of PKM-093 and PKM-883, there is still a chance that these bands are shown because of the non-specific binding of the antibody. To further validate whether the bands we identified are encoded by PKM-093 and PKM-883, we used three different siRNAs to inhibit the expression all protein isoforms of *PKM*. As shown in Figure 4d and Figure S4, the cellular *PKM* level is decreased with the siRNA transferred to the cells. In addition, we found the bands located at 49 kDa and 40 kDa also significantly decreased. This indicated that the two bands we identified are encoded by *PKM*. Moreover, we manually cut the gel with protein in PC3 from 37 kDa to 50 kDa based on the marker and separated it into three horizontal slices. Subsequently, we subjected the samples to enzymatic digestion and extracted peptides for analysis in mass spectrometry (MS). Consequently, we detected signals of peptides from PKM-093 and PKM-883 on both the top (49.9 kDa) and bottom (40.2 kDa) slices (Table S16). As shown in Figure 4e, both of these slices showed high MS intensity with good peptide coverage, proving that the bands we identified are related to the corresponding transcripts. With respect to the one from PKM-609, we could not visually separate the bands from PKM1 and PKM2 since it had a similar mass compared to PKM1 and PKM2. 

### 2.7. Exploring the Biological Function of the Prognostic Transcripts

**Figure 5:**
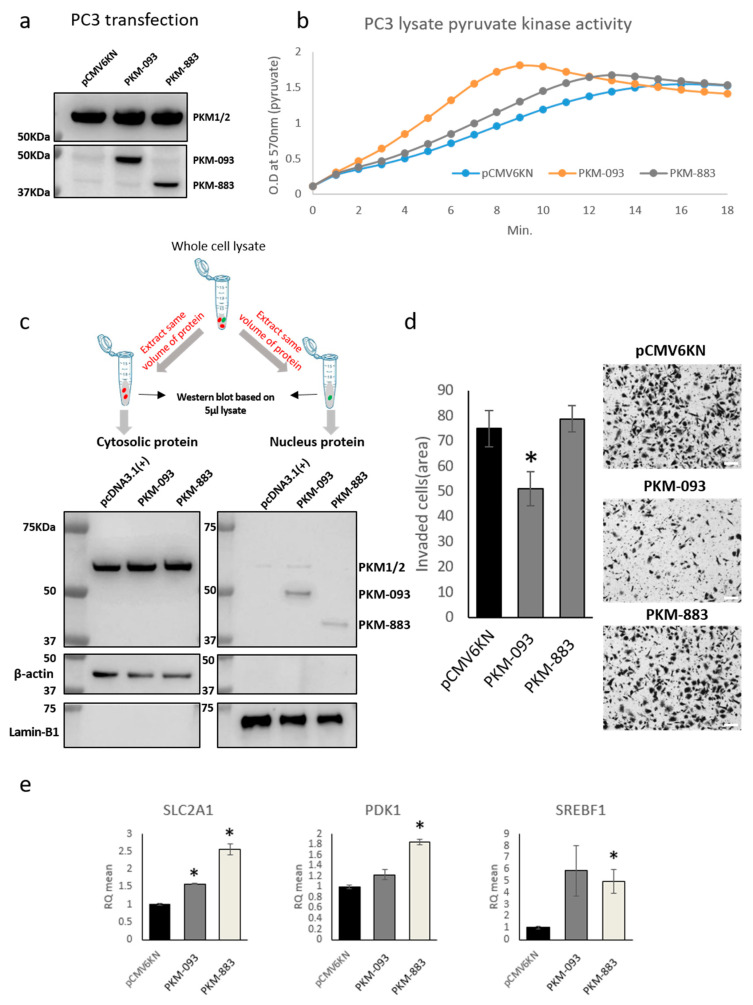
Biological effect of PKM transcript over-expression in PC3 human prostate cancer cell line. (**a**) Western blots image showing the over-expression of transfected PKM-093 and PKM-883, respectively. (**b**) Pyruvate kinase activity kinetic assay. Kinetic PK activity was measured using 7.5 µg of each transfected cell lysate. PKM-093 and PKM-883 reached faster plateau status at 6 and 8 min, respectively, than pCMV6KN empty vector control which reached plateau at 10 min. (**c**) Western blots for the overexpressed proteins of PKM-093 and PKM-883 in cytosol and nucleus with the same protein volume (see Method Section 4.7). The red dot means cytosolic protein and the green dot means nuclear protein. (**d**) Bar chart and microscopy images showing the over-expression of PKM-093 and PKM-883 significantly inhibited cell invasion compared to negative control. Error bars indicate standard errors. * indicates *t*-test *p* < 0.05. Scale bar = 100 micrometer (µm). (**e**) Quantitative PCR analysis showing the mRNA level changes of SLC2A1, PDK1, and SREBF1 in PC3 cells with PKM-093 and PKM-883 over-expression compared to negative control. Error bars indicate standard errors. * indicates *t*-test *p* < 0.05.

In order to explore the function of the prognostic transcripts identified in this study, we designed vectors and transfected to PC3 cancer cell lines to over-express PKM-093 and PKM-883. We excluded PKM-609 since the stop codon is missing in the genome annotation. As shown in Figure 5a and Figure S5, we observed that the protein levels of PKM-093 and PKM-883 were elevated compared to negative control, which proved that the transfection is successful as expected. We did not observe any significant changes in cell viability after the over-expression of the transcripts (Figure S6). To test whether over-expression of the transcripts increases the pyruvate kinase activity, we extracted the cell lysate from PC3 cell lines with empty vector, PKM-093 over-expression, and PKM-883 over-expression and found that the enzymatic activity is significantly increased in cell lysate with the overexpressed transcripts compared to negative control (Figure 5b). We also repeated the same experimental procedure using U2OS cancer cell lines and obtained similar results (Figure S7). Moreover, we used another vector to overexpress PKM-093 and PKM-883 with no tag, HA tag at the C-terminal, and FLAG tag at the N-terminal, and respectively evaluated the pyruvate kinase activities in their cell lysates. Interestingly, we observed that overexpressed PKM-093 and PKM-883 with no tag and tag at the N-terminal but not C-terminal could elevate the pyruvate kinase activity in the cell lysate (Figure S8). This suggested that the tag in the C-terminal changed the C-C interface structure, thus disturbing the formation of tetramer which is the enzymatically more activated form compared to dimer. This strongly indicated that the PKM-093 and PKM-883 are functional and protein products of these transcripts possess the pyruvate kinase activity. 

To assess the relative abundance of PKM-093 and PKM-883 in cell cytosol and nucleus, we overexpressed these two proteins and extracted cytosolic and nucleic protein from the same cell portion in order to compare their abundance by western blot using PKM antibody. Surprisingly, we observed that the majority of the overexpressed PKM-093 and PKM-883 proteins are mainly located in the nucleus, which is the opposite compared to PKM1/2 (Figure 5c and Figure S9). This suggests that PKM-093 and PKM-883 may have different functional purposes compared to PKM1/2 and play important regulatory roles. In addition, we evaluated the effect of over-expression on cell invasion using an experimental assay. As shown in Figure 5d, we observed that the cells with PKM-093 over-expression exhibited significantly inhibited invasion (~30% decrease compared to negative control), while the over-expression of PKM-883 had no significant effect on cell invasion of PC3 which is a PRAD cell line. This is in good agreement with what we observed for PKM-093 since cell migration has been negatively associated with PKM-093 in differential expression analysis in PRAD patients. To further investigate the functional role of these two transcripts, we quantified the mRNA expression of several important marker genes in negative control, PKM-093 and PKM-883 overexpressed PC3 cancer cell lines using qPCR (Figure 5e). We found that the over-expression of both transcripts significantly increased the expression of GLUT-1 (SLC2A1), which is the main glucose transporter. Our results indicated that these two transcripts may play important roles in glucose metabolism as we expected. *PDK1* is an important gene in hypoxia and it has been reported to be associated with the abnormal expression of PKM2 and Warburg effect [47]. We also observed that the expression of *PDK1* has been significantly elevated with over-expression of PKM-883. The change of *PDK1* indicated a potential role of PKM-883 in hypoxia response. *SREBF1* is a well-known transcription factor that regulates fatty acid metabolism, and it has been reported that the downregulation of PKM2 reduced the expression of *SREBF1* in cancer cells [48]. In our analysis, *SREBF1* is also significantly increased with PKM-883 over-expression, suggesting PKM-883 has potential regulatory function in fatty acid metabolism. Based on these results, we concluded that protein products of both PKM-093 and PKM-883 possess pyruvate kinase activity in different cancer cell lines and these transcripts may have a major role in biological functions associated with cancer progression.

## 3. Discussion

Protein isoforms produced by alternative splicing of the same gene contribute to proteome complexity and modulate the patterns of gene expression that govern many cell fate decisions [49]. There are seven basic alternative splicing events including exon skipping, mutually exclusive exons, alternate acceptor sites, alternate donor sites, alternate promoter, alternate terminator, and retained intron. Previous studies have already detected the survival-associated alternative splicing events or differentially splicing events involved in specific exons as the prognostic biomarker or drug response biomarker in different human cancers [50,51,52,53]. It has also been reported that the different alternative splicing events occurring in the same gene show opposite prognostic effects in the same cancer type [50,51], suggesting that these protein isoforms may have diverse or even opposite biological functions [54]. In this study, we only focus on the expression of different alternatively spliced transcripts of *PKM* and explore the potential biology functions and prognostic effects in different cancers. Several studies have been performed for studying the functional role of *PKM* in cancer metabolism, mainly focusing on PKM1 and PKM2 isoforms. With the development of bioinformatics tools for the analysis of the RNA-seq data like Kallisto [55], it is now possible to quantify the isoforms and perform systematic studies for revealing the functional role of transcripts in cancer progression. Here, we performed a transcript level analysis of *PKM* and found that four of them, including PKM2, PKM-609, PKM-093, and PKM-883, could play a key role in KIRC progression. We found that mRNA expression of these four transcripts exhibited opposite survival correlation. For example, the over-expression of PKM2 and PKM-609 indicates the favorable survival outcome for KIRC patients while the over-expression of PKM-093 and PKM-883 indicates unfavorable survival outcome. In contrast, the prognostic effect of PKM2 compared with PKM-093 and PKM-883 are reversed in CESC, PAAD, and BRCA. Moreover, we found that these isoforms of *PKM* potentially regulate the same set of biology functions with opposite regulatory directions based on the systems biology analysis. These results might explain the controversial prognostic indication of *PKM* in different cancers reported previously. Next, we validated their prognostic effect in an independent KIRC cohort, which provides additional confidence of our prognostic finding. To further support our bioinformatic prediction, we characterized for the first time the protein products of these key transcripts using western blots and mass spectrometry-based proteomics analysis and demonstrated that these proteins possess pyruvate kinase enzyme activity and could potentially play important roles in glucose and lipid metabolism, invasion, and hypoxia.

Previous studies reported that the ratio between PKM1 and PKM2 isoforms plays a key role in cancer progression [56,57,58,59,60]. In our study, we found PKM1 is not strongly associated with the survival of cancer patients as has been reported [34]. Based on our analysis, we observed that the disagreement between the studies may be explained by the differences in transcriptomic quantification methods used in the analysis of the data. For instance, a recent study quantified the mRNA level of PKM1 and PKM2 using RT-PCR and reported their association in cancer [61], but the primer they have used for RT-PCR could also bind to PKM-883 and PKM-609. Thus, the RNA level suggested for PKM1 transcript also includes other *PKM* transcripts rather than just PKM1. In addition, we also found that the RNA level of PKM1 (<5%) is very low compared to PKM2 (~95%) which is in good agreement with proteomics data reported earlier [62], and it has the same order of magnitude as PKM-883 and PKM-609 (Table S2). In this context, it is very likely that PKM-883 or PKM-609, which showed prognostic effect in our study, may also play a key role in tumor metabolism. 

We also showed the protein products of these two transcripts using western blots and validated their presence by MS after the identification of functional alternatively spliced *PKM* transcripts. It is quite difficult to distinguish these transcripts and PKM2 since they shared the majority of their nucleotide and AA sequences. For instance, the antibody we used in this study is designed to specifically target the PKM2 protein, but it also bound the protein products of PKM-883 and PKM-093. Therefore, this might be a potential explanation for the contradicting prognostic effect related to PKM2 in different studies, and there may be a need to revisit some of the previous studies to investigate all isoforms of *PKM*. 

## 4. Materials and Methods

### 4.1. Data and Preprocessing

The TCGA transcript-expression level profiles (TPM and count values) of 25 cancer types with more than 100 patients—excluding low grade glioma (LGG) for the same reason as in our previous study [3]—were downloaded from https://osf.io/gqrz9 [63] on 27 November 2018. We only extracted the tumor samples with sample identifier of BRC patient barcode ‘01′ for solid tumor and ‘03′ for acute myeloid leukemia, respectively, and with the vial identifier “A” for all the tumor types. We quantified the mRNA expression using Kallisto [55] based on the GENCODE reference transcriptome (version 24) (Ensembl 83 (GRCh38.P5)). The clinical information of TCGA samples was downloaded through R package TCGAbiolinks [64]. The whole-exome sequences data of 100 KIRC samples of patients from Japanese cohort [43] were download from European Genome-phenome Archive (accession number: EGAS00001000509). BEDTools [65] was used for converting BAM to FASTQ file. Kallisto was used for estimating the count and TPM values of transcripts based on the same reference transcriptome of TCGA data. The sum value of the multiple transcripts of a gene was used as the expression value of this gene. The genes with average TPM values >1 across patients in each cancer were analyzed. 

### 4.2. Survival Analysis

Based on the TPM value of each transcript or gene, we classified the patients into two groups and examined their prognoses. Survival curves were estimated by the Kaplan–Meier method and compared by log-rank test. To choose the best TPM cutoffs for grouping the patients most significantly, all TPM values from the 10th to 90th percentiles used to group the patients, significant differences in the survival outcomes of the two groups were examined and the value yielding the lowest log-rank *p*-value was selected. The R package survival and graphics were used for survival analysis and plotting survival curves.

For retrieving prognostic signature, we used the expression cutoff obtained in the individual survival analysis for each of the four transcripts which could classify the patients into two groups with significantly different prognoses. In TCGA cohort, if the expression of PKM2 or ENST00000561609 was less than 476.35 or 0.69 in a sample, this sample was classified into high-risk group, otherwise, low-risk group. On the other hand, if the expression value of ENST00000389093 or ENST00000568883 was higher than 18.18 or 13.74 in a sample, this sample was classified into high-risk group, otherwise, low-risk group. Similarly, the cutoffs of the four transcripts were 815.84, 0.33, 7.90, and 6.63 in the Japanese cohort. Therefore, we classified the samples of the two different cohorts into the high-risk group when at least two transcripts were higher or lower than the corresponding cutoffs.

Moreover, univariate and multivariate Cox analyses were performed to confirm the prognostic values of these four transcripts based on R package ‘survival’. We took the expression of PKM1/2 as a covariate when we evaluated the prognostic values of the other transcripts in multivariate Cox analysis because of the sequence similarity. 

### 4.3. Differential Expression Analysis

DESeq2 [66] was used to identify differentially expressed genes (DEGs) between two groups. Before performing DESeq2, we removed the low expressed genes with average TPM ≤ 1 and then we used the raw count values of the rest genes as the input of DESeq2. The Benjamini–Hochberg procedure was used to estimate FDR. FDR < 1.0 × 10^−5^ was used to identify significant DEGs.

### 4.4. Overlapping of Two Lists of Differentially Expressed Genes DEGs

If DEG list 1 with *L*_1_ genes and DEG list 2 with *L*_2_ genes have *k* overlapping genes, among which *s* genes shows the same directions (up- or down-regulation) in the two DEGs lists, the probability of observing at least *s* consistent genes by chance can be calculated according to the following cumulative hypergeometric distribution model:

$$P=1-\overset{s-1}{\underset{i=0}{\sum }}\frac{\left(\begin{array}{c} L_{2}\\ i \end{array}\right)\left(\begin{array}{c} L-L_{2}\\ L_{1}-i \end{array}\right)}{\left(\begin{array}{c} L\\ L_{1} \end{array}\right)}$$

where *L* represents the number of the background genes commonly detected in the datasets from which the DEGs are extracted. The two DEG lists were considered to be significantly overlapped if *p*  <  0.05.

The concordance score *s*/*k* is used to evaluate the consistency of DEGs between the two lists. Obviously, the score ranges from 0 to 1, and the higher concordance score suggested the better consistency of two lists of DEGs.

### 4.5. Functional Enrichment Analysis

Gene ontology (GO) enrichment was performed by the enrichGo function in R package ClusterProfiler [67], in which the hypergeometric distribution was used to calculate the statistical significance of biological pathways enriched with DEGs of interest. 

### 4.6. Hierarchical Clustering 

Log-rank *p*-values were hierarchically clustered by Spearman correlation distance and Ward linkage method (ward.D2). Negative log 10 transformation of *p*-values was performed before clustering. 

### 4.7. Western Blots

Whole cell lysate was extracted with CelLytic M (C2978, Sigma-Aldrich, Saint Louis, MO, USA) lysis buffer. Cytosolic and nucleus protein was extracted with Nuclear Extraction Kit (ab113474, abcam, Cambridge, UK). Three million cells were counted and collected. Fifty micrograms of whole lysate, cytosolic and nucleus protein were used to detect endogenous ENST00000389093 and ENST00000568883. Proteins were separated by Mini-PROTEAN^®^ TGX™ Precast Gels (Bio-Rad, Berkeley, CA, USA) and transferred using Trans-Blot^®^ Turbo™ Transfer System (Bio-Rad, Berkeley, CA, USA). PKM2 antibody (ab137791, abcam) was used for primary antibody overnight. Goat Anti-Rabbit HRP (ab205718) was blotted for one hour. PKM band were detected with ImageQuanattm LAS 500 (29-0050-63, GE) automatic exposure procedure and two PKM isotype band (49.9 kDa and 40.2 kDa) were detected for 5 min exposure. Band intensity was measured via ImageJ program. Overexpressed ENST00000389093 and ENST00000568883 localization was observed by western blot analysis. Cytosolic and nucleus protein lysate were prepared with Nuclear Extraction kit (ab113474). After cytosolic lysate extraction with 80 µL pre-extraction buffer, insoluble nucleus had been washed with 150 µL of pre-extraction buffer and extracted whole nuclear lysate with 80 µL of ENE2 (ab113474) nuclear extraction buffer with sonication (Diagenode Bioruptor 300). Sonication was performed as 30 s sonication, 30 s rest for 20 cycles at high frequency. Five microliters of the same volume cytosolic and nucleus lysate were loaded and analyzed with western blot. PKM2 antibody (ab137791, abcam) Lamin B1 (ab16048) beta-actin (ab8227) antibody were blotted for 1.5 h. Goat Anti-Rabbit HRP secondary antibody was blotted for 1 h. Cytosolic and nucleus FLAG tagged ENST00000389093 and ENST00000568883 were detected in the same transfer membrane with ImageQuanattm LAS 500 1-min exposure.

### 4.8. Cell Culture and siRNA Transfection

All cells were cultured followed by ATCC instruction. PC3 cell culture media formulation is F12K Nutrient mix supplemented with 10% FBS and 1% Penicillin/Streptomycin. U2OS cells were cultured with McCoy’s 5A Medium with 10% FBS and 1% Penicillin/Streptomycin, MRC5 cells were cultured with DMEM with 10% FBS and 1% Penicillin/Streptomycin, and RWPE-1 cells were cultured with Keratinocyte Serum Free Medium (K-SFM) supplemented with Bovine Pituitary Extract (BPE) and human recombinant Epidermal Growth Factor (EGF) (Kit Catalog Number 17005-042). For siRNA treatment, 400,000 cells were seeded to six-well plate. After 24 h of cell seeding 25 pmol siRNA was transfected by Lipofectamine^®^ RNAiMAX (13778-075 Invitrogen) for two days. pCMV6KN empty vector from ORIGENE and pcDNA3.1(+) empty vector from GeneScript were used to clone ENST00000389093 and ENST00000568883 transcripts. Three hundred thousand cells of PC3 were seeded to a six-well plate and FuGENE^®^ HD (E2311 Promega) was used to transfect cloned vector as 1:3 (3 µg of DNA, 9 µL of FuGENE^®^). After two-days transfection, culture media were changed to the fresh media. RNA and protein lysate were harvested at the third day.

### 4.9. Sample Preparation for Mass Spectrometry Analysis

The gel pieces were subjected to in-gel digestion as described in previous study [68], with some adjustments. Reduction was performed by addition of 10 mM dithiothreitol and incubation at 56 °C for 30 min. The samples were alkylated by addition of 55 mM 2-chloroacetamide and incubation shielded from light for 20 min at room temperature. Tryptic digestion was performed overnight at 37 °C after addition of trypsin solution containing 13 ng/µL proteomics grade porcine trypsin (Sigma Aldrich, St Louis, MO, USA), 100 mM ammonium bicarbonate, 10% acetonitrile (ACN). The peptides were then extracted by addition of 100 µL extraction buffer to each sample (1:2, 5% formic acid (FA)/ACN). The extracted peptides were transferred to HPLC-vials and dried using vacuum centrifugation. The peptides were then resuspended in 60 µL 3% ACN, 0.1% FA and analyzed by liquid chromatography (LC)-MS/MS.

### 4.10. MS Analysis

PC3 cell lysate was prepared with CelLytic M (C2978, Sigma-Aldrich) lysis buffer. SDS PAGE separated 60 µg of PC3 cell lysate per well with Precision Plus Protein Standards ladder (1610374, Bio-Rad). Gel pieces were cut by razor blade, three pieces between 37 and 50 kDa ladder indicated. 

The samples were analyzed using a Thermo Scientific Q Exactive HF (Thermo Fisher Scientific, Waltham, MA, USA) online connected to a Dionex Ultimate 3000 UHPLC-system (Thermo Fisher Scientific) equipped with a reverse phase trap column (Acclaim PepMap 100, 75 μm × 2 cm, 3 μm, 100 Å; Thermo Fisher Scientific) and 50 cm analytical column (EASY-Spray, 75 µm × 50 cm, 2 μm, 100 Å; Thermo Fisher Scientific). Ten microliters of each sample was injected for analysis and the peptides were separated over an 85 min run using a 60 min linear LC-gradient and sprayed directly into the mass spectrometer using the EASY-Spray ion source. The solvents used for the LC-gradient were 3% ACN, 0.1% FA (solvent A) and 95% ACN, 0.1% FA (solvent B). The flow rate of the system was set to 300 nL/min and the gradient used was as follows: 5% solvent B for 3 min, 5–35% solvent B within 60 min, 35–90% solvent B within 5 min, 90% solvent B for 7 min, 90–5% solvent B within 0.1 min, 5% solvent B for 10 min. The mass spectrometer was set to operate using a Top10 MS method with a full scan resolution of 60,000 (mass range: 400–1200 *m*/*z*, AGC: 3 × 10^6^) and a MS/MS resolution of 30,000 (AGC: 1 × 10^5^). The normalized collision energy was set to 30.

### 4.11. Data Analysis of MS Results

The raw files obtained from the MS experiment were analyzed using MaxQuant (version 1.6.1.0) [69] implementing Andromeda [70] to search the MS/MS data against the Ensembl Homo sapiens database (version 83.38, all protein coding transcripts from the primary assembly) as well as a separate database with the two distinct target sequences (ENST00000389093 and ENST00000568883) a list of common contaminants. Trypsin/P was used for cleavage specificity with up to two missed cleavages. Oxidation (M) was used as a variable modification while carbamidomethylation (C) was used as a fixed modification. The peptide and protein FDR were set to 1% and the minimum peptide length was set to seven amino acids. The presence of the target proteins was assessed by evaluating the identification of unique peptides specific to the proteins in the different samples.

### 4.12. Homology Model

The homology models were built using StructurePrediction panel in Schrödinger Suite (Schrödinger, LLC., New York, NY, USA). The ClustralW method was used to align the target and template sequences in Prime, the energy-based was selected for model building method, and homo-multimer was selected for multi-template model type. The homology model of ENST00000561609 was built based on the PKM2 crystal structure (PDB ID: 5X1W), as ENST00000561609 shares 96% sequence similarity to PKM2, compared to 91% to PKM1. ENST00000389093 and ENST00000568883 share higher sequence similarity to PKM1, with 100% and 92% correspondingly. These two homology models were built based on the PKM1 crystal structure (PDB ID: 3SRF).

### 4.13. Quantitative PCR (qPCR) Analysis of SLC2A1, PDK1, SREBF1, SNAI2, FN1, and CASP7 Expression

Cloned vector transfected PC3 cells were prepared as biological and technical triplicate. RNA was isolated using TRIzol^®^ Reagent (15596018, Life Technologies Carlsbad, Carlsbad, CA, USA), and RNeasy^®^ Plus Mini Kit (74136 Qiagen, Hilden, Germany) following the manufacturer’s instructions. One microgram of total RNA was reverse transcribed into cDNA using GoScriptTM Reverse Transcription System (A5001, Promega, Madison, WI, USA) with manufacturer’s guidelines. CFX ConnectTM Real-Time System (Bio-Rad, Hercules, CA, USA) used to measure transcript level of target genes. iQ SYBR^®^ Green Supermix (1708882, Bio-Rad) was used to PCR mixture. Just 0.5 µL of cDNA and 1 µL of 100 nM each primer was mixed to 20 µL total mixture. qPCR program has consisted of 45 cycles of denaturation for 10 s at 95 °C, elongation, and detection for 30 s at 60 °C. When that final cycle completed, melting curve analysis was performed within range of 55–95 °C (10 s, 0.5 °C). Final mRNA expression was calculated as average CQ value of biological and technical triplicates. Primer sequences are Beta actin: CACCAACTGGGACGACAT (Forward) and ACAGCCTGGATAGCAACG (Reverse), SLC2A1: CCACTGCAACGGCTTAGACTT (Forward) and TGGGTAACAGGGATCAAACAGA (Reverse), PDK1: CCTGGACTTCGGATCAGTGA (Forward) and TGCCGCAGAAACATAAATGA (Reverse), SREBF1: GCTCCTCCATCAATGACAAAATC (Forward) and TGCAGAAAGCGAATGTAGTCGAT (Reverse), SNAI2: AGCTACCCAATGGCCTCTCT (Forward) and TCACTCGCCCCAAAGATG (Reverse), FN1: CCAAGAAGGGCTCGTGTGA (Forward) and GGCTGGAACGGCATCAAC (Reverse), CASP7: GCCTGGGTTTTGACGTGATT (Forward) and CAGGCGGCATTTGTATGGT (Reverse).

### 4.14. Pyruvate Kinase Activity Assay 

Whole cell lysate was prepared with lysis buffer (C2978, Sigma-Aldrich). Three micrograms of PC3 cell whole lysate was used for pyruvate kinase assay (ab83432, abcam) following manufacturer’s instruction. Kinetic measurement at O.D 570 nm by microplate reader (Hidex Sense Beta Plus) recorded pyruvate kinase activity every minute for 40 min.

### 4.15. Invasion Assay

Cloned vectors were transfected to 300,000 cells of PC3. Day 2 transfection, trypsinize cells and seed 200,000 cells to collagen coated 8 µm pore trans-well insert (3422, CORNING) with serum free media. Trans-well placed to 600 µL of growth media in 24-well plate, then growth factors in growth media induced invasion to bottom. After 24-h, trans-wells were washed with PBS and stained by 0.2% crystal violet solution. PBS immersed cotton swab removed PC3 cells not invaded on upper well. Six images for invaded cells were taken from middle, left, right, top, bottom, and right top of bottom side of trans-well. Stained cells were counted and plotted as histogram.

## 5. Conclusions

We discovered the potential functional and prognostic roles of the three alternatively spliced *PKM* transcripts in KIRC and different cancers based on systems biology analysis, and partly validated them with in vitro experiments. Our study could serve as a primer for future studies focusing on revealing the biological and oncological role of these alternative spliced variants of *PKM*. 

## Data Availability

Publicly available datasets were analyzed in this study. This data can be found here: https://osf.io/gqrz9.

## Supplementary Materials

The following are available online at https://www.mdpi.com/2072-6694/13/2/348/s1, Figure S1: Overlapping of DEGs between transcripts in TCGA KIRC cohort, Figure S2: Homology modelling predicted structures of the protein products of PKM-609, PKM-093, and PKM-883, Figure S3: The uncropped and cropped western blot images showing the proteins encoded by the transcript PKM-093 and PKM-883, Figure S4: The uncropped and cropped western blot images showing the protein level of PKM1/2, PKM-093, and PKM-883 with siRNA and negative control, Figure S5: The uncropped and cropped western blots showing the over-expression of transfected PKM-093 and PKM-883, respectively, Figure S6: Cell viability of PC3 and U2OS cells with empty vectors, PKM-093 over-expression, and PKM-883 over-expression, Figure S7: Pyruvate kinase activity in U2OS cells with empty vectors, PKM-093 over-expression, and PKM-883 over-expression, Figure S8: Pyruvate kinase activity in PC3 cells with overexpressed PKM-093 and PKM-883 with no tag, HA tag at C-terminal, and flag tag at N-terminal, Figure S9: The uncropped western blot images showing the overexpressed proteins of PKM-093 and PKM-883 in cytosol and nucleus within the same cell portion, Table S1: The start and end sites of exons and introns of PKM transcripts, Table S2: The average expression of PKM and 14 *PKM* transcripts in 25 cancer types, Table S3: Log-rank *p*-value of PKM and its transcripts in 25 cancer types, Table S4: The adjusted log-rank *p*-value of PKM and its transcripts in each of the 25 cancer types based on BH method, Table S5: The enriched of GO terms with DEGs for all transcripts in 25 cancer types, Table S6: Overlapping of DEGs between transcripts in KIRC for *PKM* transcripts, Table S7: Overlapping of DEGs between transcripts in CESC for *PKM* transcripts, Table S8: Overlapping of DEGs between transcripts in PAAD for *PKM* transcripts, Table S9: Overlapping of DEGs between transcripts in BRCA for *PKM* transcripts, Table S10: Overlapping of DEGs between transcripts in COAD for *PKM* transcripts, Table S11: Univariate and multivariate Cox analysis of four transcripts in TCGA KIRC cohort, Table S12: Overlapping of DEGs between TCGA and Japanese cohorts for four *PKM* transcripts, Table S13: The enriched GO terms for the DEGs between high- and low-risk groups identified from TCGA and Japanese cohorts, Table S14: The alignment of amino acid sequences encoded by PKM1, PKM2, PKM-609, PKM-093, and PKM-883, Table S15: Relative protein level between protein products of PKM-093 and PKM-883 compared to PKM1 plus PKM2, Table S16: The intensity of peptides in mass spectrometry.
