# Supplementary material for: Discovery of Functional Alternatively Spliced PKM Transcripts in Human Cancers
Source: Cancers (Basel). 2021 Jan 19;13(2):348. doi: 10.3390/cancers13020348 (PMC7835739; doi:10.3390/cancers13020348)
Supplement: Supplementary file 1 [file cancers-13-00348-s001.zip › Supplementary_Figures.pdf]

Supplementary Materials

Discovery of Functional Alternatively Spliced *PKM* Transcripts in Human Cancers

Xiangyu Li, Woonghee Kim, Muhammad Arif, Chunxia Gao, Andreas Hober, David Kotol, Linnéa Strandberg, Björn Forsström, Åsa Sivertsson, Per Oksvold, Hasan Turkez, Morten Grøtli, Yusuke Sato, Haruki Kume, Seishi Ogawa, Jan Boren, Jens Nielsen, Mathias Uhlen, Cheng Zhang and Adil Mardinoglu

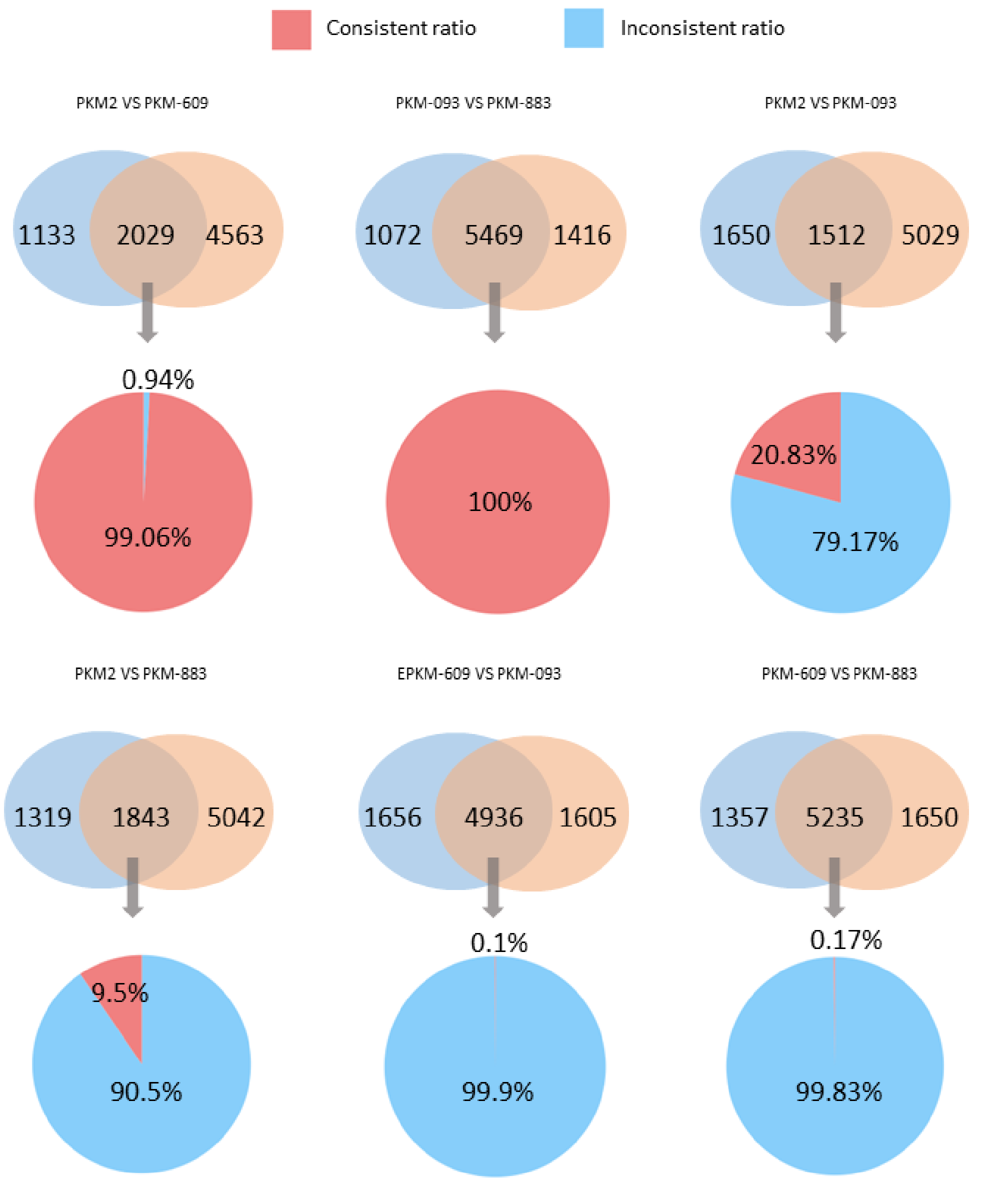

Figure S1. Overlapping of DEGs between transcripts in TCGA KIRC cohort.

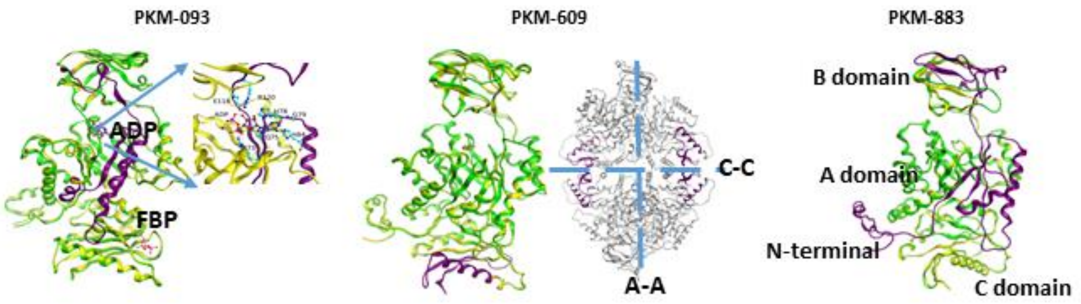

Figure S2. Homology modelling predicted structures of the protein products of PKM-609, PKM-093 and PKM-883.

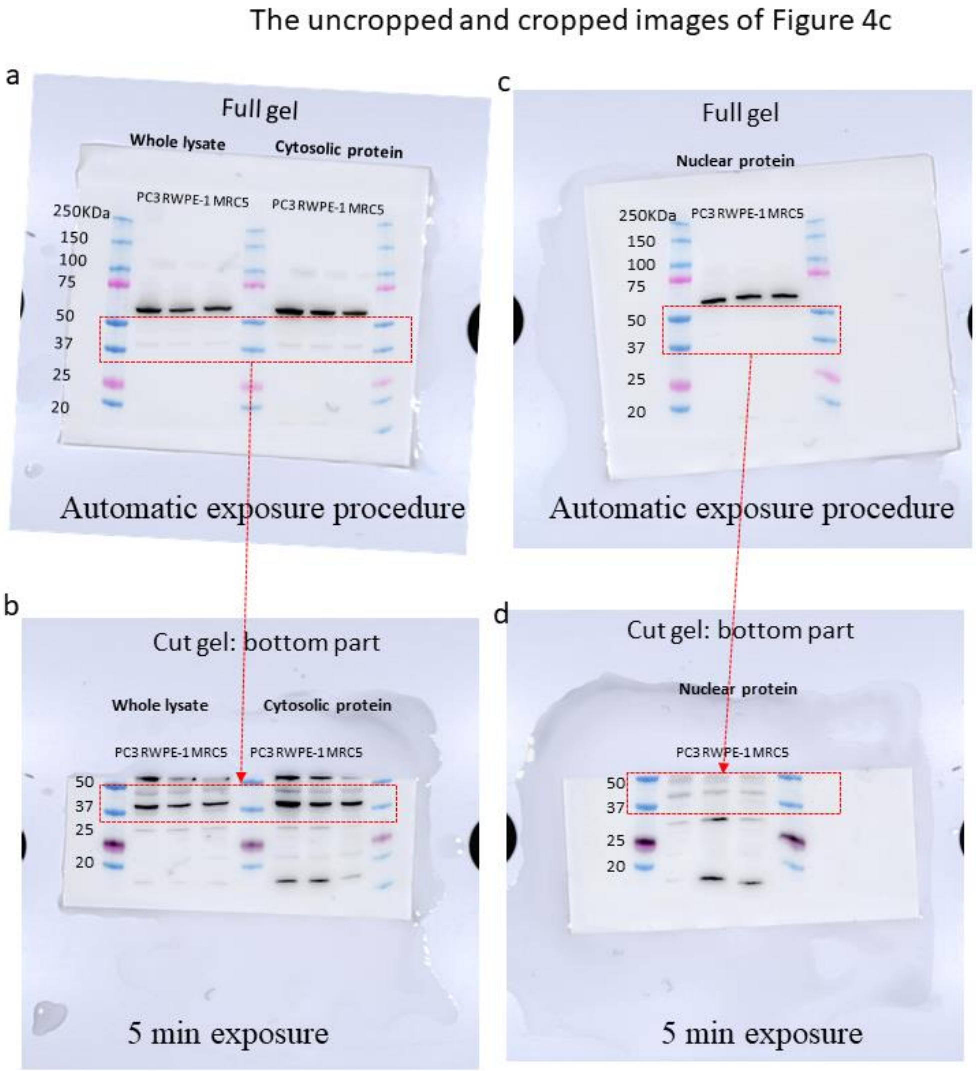

Figure S3. The uncropped and cropped Western blot images showing the proteins encoded by the transcript PKM-093 and PKM-883.

The uncropped and cropped images of Figure 4d

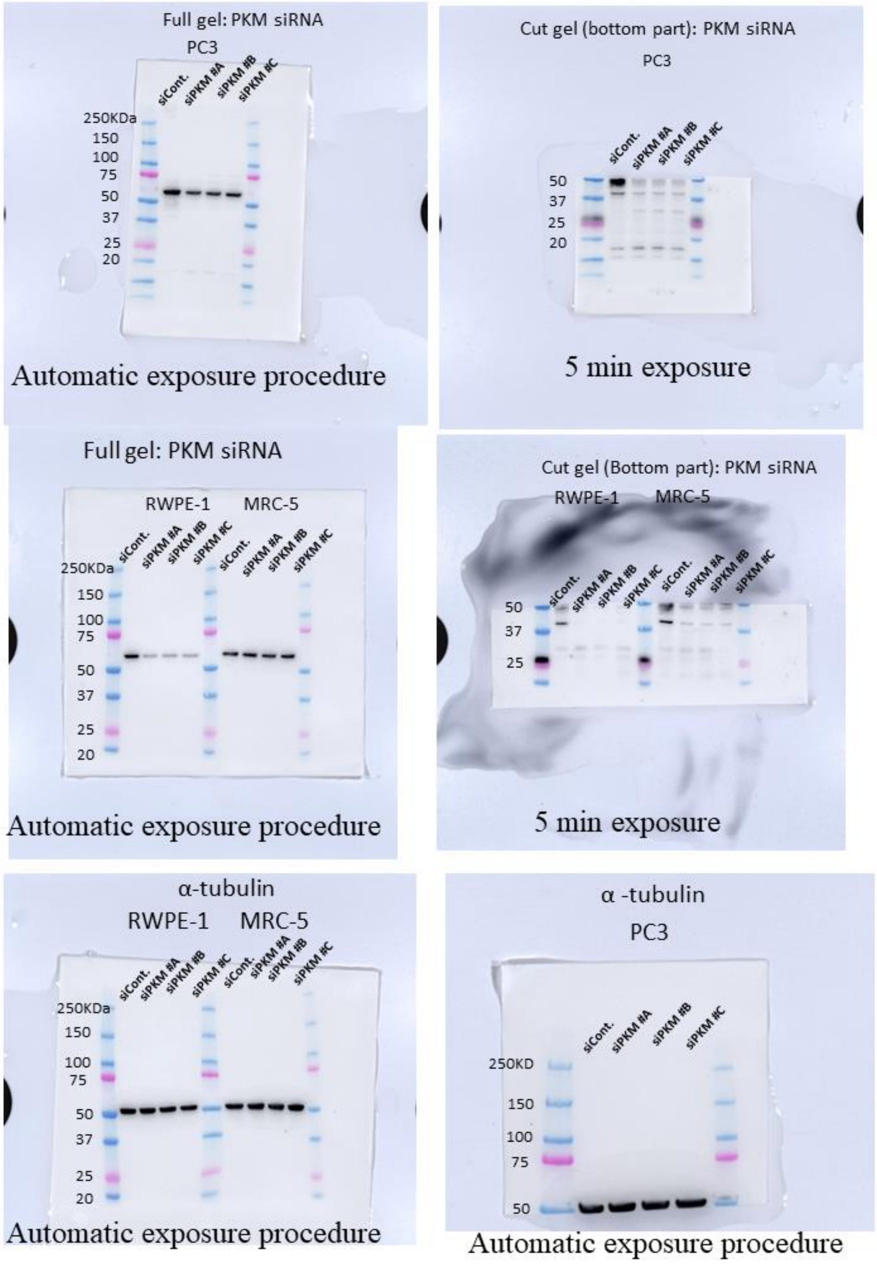

Figure S4. The uncropped and cropped Western blot images showing the protein level of PKM1/2, PKM-093 and PKM-883 with siRNA and negative control.

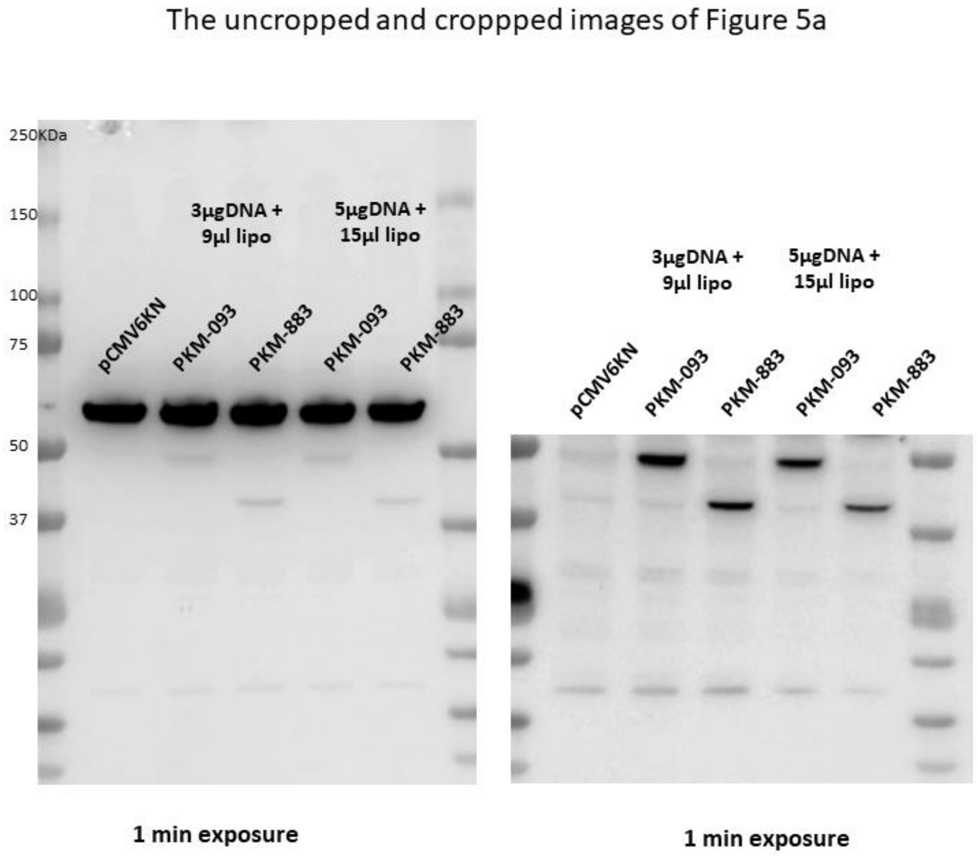

Figure S5. The uncropped and cropped Western blots showing the over-expression of transfected PKM-093 and PKM-883, respectively.

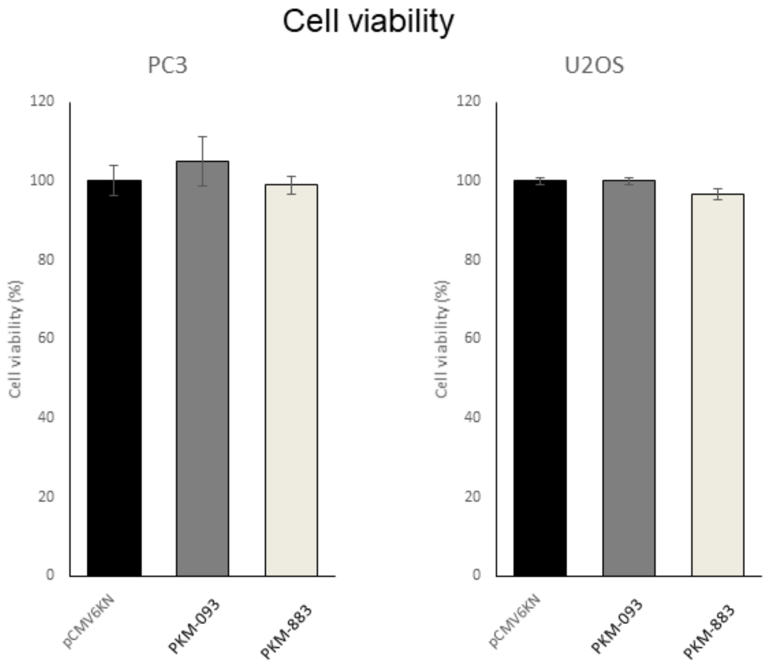

Figure S6. Cell viability of PC3 and U2OS cells with empty vectors, PKM-093 over-expression and PKM-883 over-expression.

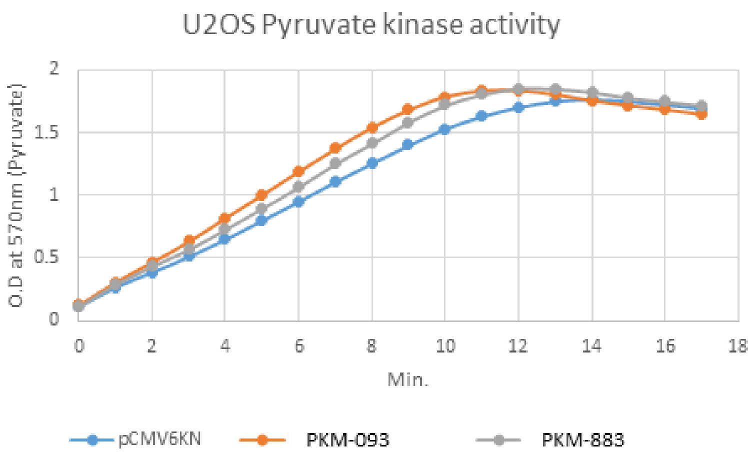

Figure S7. Pyruvate kinase activity in U2OS cells with empty vectors, PKM-093 over-expression and PKM-883 over-expression.

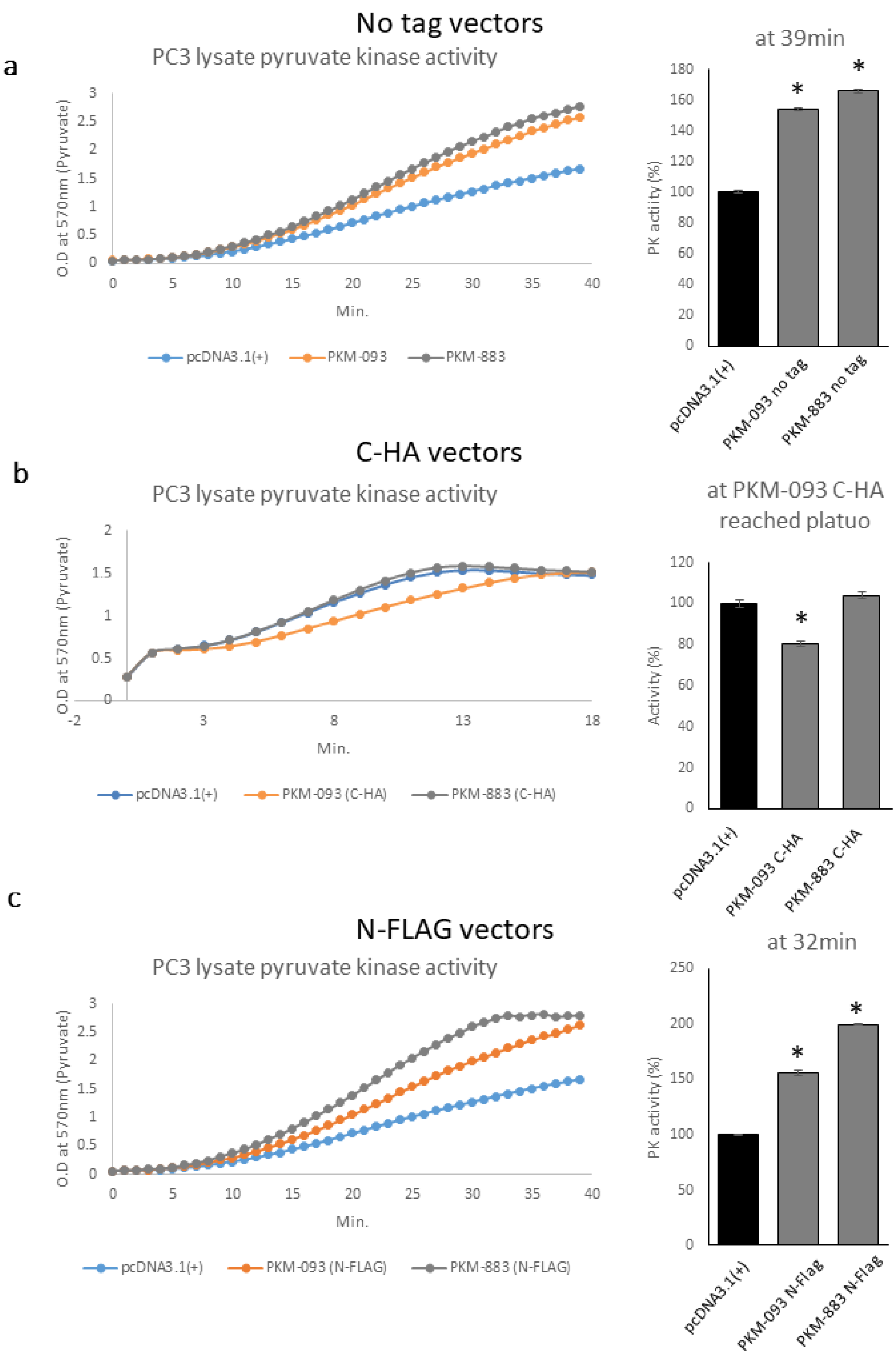

Figure S8. Pyruvate kinase activity in PC3 cells with overexpressed PKM-093 and PKM-883 with no tag, HA tag at C-terminal and flag tag at N-terminal.* indicates *t*-test $p < 0.05$.

The uncropped images of Figure 5c

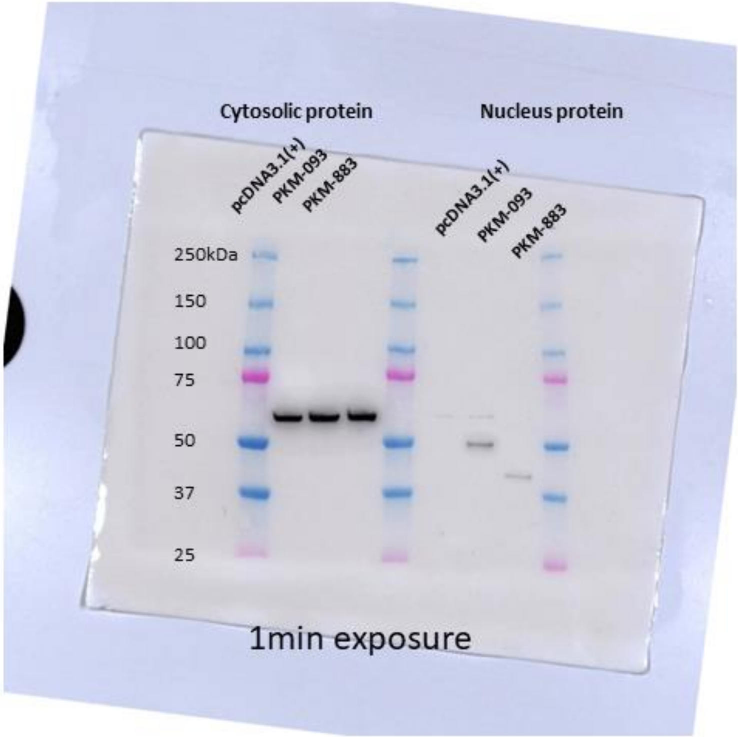

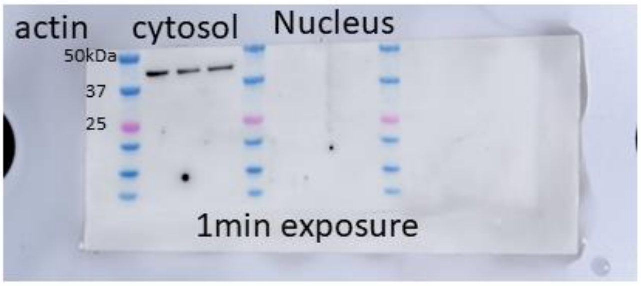

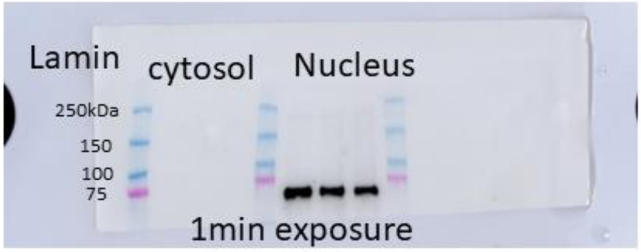

Figure S9. The uncropped Western blot images showing the overexpressed proteins of PKM-093 and PKM-883 in cytosol and nucleus within the same cell portion.

© 2021 by the authors. Licensee MDPI, Basel, Switzerland. This article is an open access article distributed under the terms and conditions of the Creative Commons Attribution (CC BY) license (http://creativecommons.org/licenses/by/4.0/).
